# 

**Published:** 1895-08-17

**Authors:** 


**Notices of Births, Deaths, and Marriages** are inserted in this column at a charge of 2s. 6d. for an announcement not exceeding four lines.

**Notice to Advertisers and Subscribers.**

All Advertisements, Orders for Copies of The Hospital and Business Communications should be addressed to **The Manager, NOT TO THE EDITOR,** The Hospital, 428, Strand, London, W.C.

The Cost of Subscription, post free, is as follows:—Per week, 2½d.; per quarter, 2s. 8d.; per half-year, 5s. 3d.; per annum, 10s. 6d. Foreign:—Per Annum, 15s. 2d.; payable, in all cases, in advance.

### NOTICE TO CORRESPONDENTS.

All MS., Letters, Books for Review, and other matters intended for the Editor should be addressed The Editor, The Lodge, Porchester Square, London, W.

The Editor cannot undertake to return rejected MS., even when accompanied by stamped directed envelope.

**"Burdett's Hospital Annual," 1895,**

Sixth year of publication. 950 pp. Scarlet cloth, gilt lettered. Price 5s. A most complete guide to British, Colonial, Indian, and American Hospitals, Dispensaries, Nursing and Convalescent Institutions, and Asylums. A few copies of the "Annual" for 1894 may still be obtained on application to the Publishers, The Scientific Press (Limited), 428, Strand, London, W.C.

**NOTICE.—Vol. XVII. of "The Hospital" (October, 1894, to April, 1895), in handsome cloth binding, is on sale at the Office of this Journal. Price 6s. Cases for binding the Numbers contained in this Volume, 1s. 6d. Index to Vol. XVII., 2½d., post free.**

**The Monthly Part for August is now ready, price 6d.; by post, 8d.**

### Scraps and Gleanings.

Legacies for the year at the Birmingham and Midland Eye Hospital amount to £835.

The annual conference of the British Pharmaceutical Society has been held at Bournemouth.

At Bombay and Calcutta typhoid fever has been traced to the eating of watercress grown in polluted water.

The Queen has sent a donation of £100 to the building fund of the Royal Free Hospital, Gray's Inn Road.

A large bazaar has been held at the Curragh, with the purpose of swelling the funds of the Drogheda Memorial Cottage Hospital.

A fete on a large scale has just been held in Ersham House Park, Canterbury, in favour of the funds of the Kent and Canterbury Hospital.

A contemporary, assuming a definitely alarmist tone, now warns us of the dangers of the banknote, wherein it affirms the hidden microbe is lurking.

The cyclists' carnival at Southampton in aid of the local hospitals proved a great success. A finer carnival has never been held in the town.

The fund for the new County Hospital for Bedfordshire, which was started in February last, has within the short space of five months reached the sum of £20,258.

Two additional wards in the North Charitable Infirmary, Cork, have been formally opened. These were provided out of a bequest of £28,000 of the Viscountess Combermere.

The proposed scheme for adding a children's ward to the Dewsbury and District Infirmary has been postponed for the time being, owing to financial and structural difficulties.

The Enfield District Council having failed to secure the co-operation of either South Hornsey or Wood Green, have decided to provide a Hospital for Infectious Diseases themselves.

The consumption of meat appears to be on the increase in England. In 1882 statistics show the consumption to have been 108 per head, whilst in the three years 1891-93 it was at the rate of 119lb. per head.

The governors of St. Mark's Ophthalmic Hospital, Dublin, report that their bank account is largely overdrawn, and they are earnestly requesting the aid of the public to enable them to carry on the work of the hospital.

The annual demonstration of the clubs and friendly societies of Teddington took place recently. The receipts, which will be given towards the funds of the local cottage hospital, are estimated at about £140.

The expenditure of the Carmarthenshire Infirmary during the past few years has exceeded its income, and although there is a debt upon the institution of more than £300, it is absolutely necessary to incur further expenditure by extensive repairs to the building.

Lecturing at the Royal Institution, Prof. Ray Lankester denounced in salutary terms the use of the so-called domestic "filter." He remarked that he had found bacteria-free water, after passing through such a filter, had taken up 200,000 to the cubic centimetre.

It has been decided by the Huddersfield County Borough Council that it is imperative in the interests of public health to provide further and adequate hospital accommodation, and tenders for the erection of the same are to be received forthwith at the Birkby Fever Hospital.

The Invalids' Auxiliary to the Edinburgh Medical Missionary Society will hold an annual sale of work in November. The society, which has now been in existence 17 years, is doing a good work. The proceeds of the sales are devoted to the funds of the various medical missions.

The proposed new hospital which the Oldham Corporation intend building in the Highmoor District (Yorks) is not receiving any outside encouragement. The Saddleworth Rural District Council has for several days past been busily occupied in dealing with this question.

The committee of the Alexandra Hospital for Children with Hip Disease is making an earnest appeal to all friends and supporters of the institution to help them to raise the £9,000 required for the proposed new building, the building fund of which at the present time amounts to £3,000.

A sum of £10 is to be paid from the funds of the Wigan Infirmary with the object of commemorating within the building the memory of Nurses Lister and Cook, who lost their lives in the service of the institution. The opening of the Gidlow extension of the hospital is to be made the occasion of a public demonstration.

Founders' Day at the Surrey Convalescent Home, Seaford, was celebrated by a luncheon, at which Sir Trevor Lawrence, Bart., presided. He said that all who had been to the home as patients bore the highest testimony to the kindness with which they had been treated, and he thought that Surrey people had every reason to be gratified with the success and progress of the home.

Many French scientific papers are taking up the question of the size of Bismarck's brain, a question raised by the measurements of Schaper, the sculptor, who designed the Chancellor's statue at Cologne. These measures, anthropologically regarded, show extraordinary dimensions, the weight of the brain being 35 per cent. above the average weight.

All things considered, the committee and governors of the Metropolitan Hospital have no cause to be dissatisfied with the results of their past year's labours on behalf of the sick poor of North-east London. This year will be marked in the annals of the institution by reason of Mr. Passmore Edwards' generous offer of defraying the cost of erecting a convalescent home in connection with this hospital.

The treatment of cases of consumption, as is pointed out in the report of the National Hospital for Consumption for Ireland, near Newcastle, Wicklow, requires a special and exceptional expenditure. The diet is necessarily costly, and the "separate principle" is adopted. The committee are, therefore, asking for further funds to help in the carrying on of the work. Consumption is the most prevalent and fatal malady to which the Irish people are subject. According to the latest returns of the Registrar-General, 9,869 deaths (10 per cent., that is, of all the deaths in Ireland) occurred in the year 1893 from phthisis, in addition to 13,714 deaths from other diseases of the respiratory organs.

### The Student of Medicine.

### APPOINTMENTS.

Dr. J. P. Atkinson appointed Medical Officer for the Northern District of the Lancaster Union. Dr. W. H. Bate appointed Medical Officer of Health for the Culmstock Rural Sanitary District. J. G. Beasley, L.R.C.P.Edin., L.F.P.S.Glasg., appointed Medical Officer of Health to the Rowley District Council. A. T. Brand, M.D., C.M., appointed Certifying Factory Surgeon for the District of Driffield, East Yorks. G. A. Brown, M.R.C.S.Eng., L.S.A., appointed Medical Officer for the Workhouse and Tredegar District of the Bedwellty Union. W. Bulloch, M.D., appointed Assistant Bacteriologist in the Antitoxin Department of the British Institute of Preventive Medicine. L. C. Burrell, M.A., M.B.Cantab., M.R.C.S.Eng., L.R.C.P.Lond., appointed House Surgeon to the Suffolk General Hospital. H. J. Campbell, M.D., M.R.C.P.Lond., M.R.C.S., appointed Honorary Physician to the Bradford Infirmary. H. J. Cardale, M.B., C.M.Edin., appointed Assistant House Surgeon to the Royal Albert Hospital, Devonport. G. P. Chappel, M.B., B.C.Camb., appointed Resident Medical Officer to the City of London Hospital for Diseases of the Chest, Victoria Park. A. R. Colyer, M.R.C.S., L.R.C.P., L.D.S., appointed Assistant Dental Surgeon to the Dental Hospital of London. T. E. Dakeyne, L.R.C.P.Edin., M.R.C.S.Eng., appointed Medical Officer for the Leek District of the Leek Union. J. Dobb, L.R.C.P., L.R.C.S.Edin., appointed Medical Officer of Health for the Golborne Urban Sanitary District. B. Dyer appointed Analyst to the County of Rutland. T. J. Fletcher, M.B., C.M. Edin., appointed Medical Officer for the Castle Donington District of the Shardlow Union. Mr. H. R. Gardner, jun., appointed Assistant Medical Officer at the Workhouse of the Parish of St. Matthew, Bethnal Green. C. E. Goddard, L.R.C.P.Lond., M.R.C.S.Eng., appointed Medical Officer of Health to the Wembley Urban District Council. W. R. Grove, M.B.Camb., M.R.C.S., L.R.C.P.Lond., appointed Medical Officer to the St. Ives Union Workhouse. G. C. Hall, M.R.C.S.Eng., L.S.A., appointed Medical Officer for the Ombersley District of the Droitwich Union. H. C. Harper, M.R.C.S.Eng., L.R.C.P.Lond., appointed Medical Officer of Health for the East Stow Rural Sanitary District. F. P. Hearder, M.B., C.M.Edin., appointed Second Assistant Medical Officer to the West Riding Asylum, Wakefield. Mr. D. Hunter appointed Fourth Assistant Medical Officer to the West Riding Asylum, Wakefield. Dr. J. S. Jameson appointed Medical Officer for the Ardleigh and Great Bromley and Wise District of the Tendring Union. J. Leys, M.B., C.M.Aberd., appointed Medical Officer for the Norton District of the Leek Union. T. G. Lithgow, F.R.C.S., L.R.C.P. Lond., D.P.H.Lond., appointed Certifying Factory Surgeon for the Farnborough District. H. W. Lyle, M.R.C.S.Eng., L.R.C.P. Lond., appointed Lecturer in Animal Biology and Demonstrator of Physiology at King's College, London. S. McCoull, M.B., B.S.Durh., appointed Medical Officer for the Tenth District of the Hexham Union. S. E. Morton, M.R.C.S., L.R.C.P., appointed Senior House Surgeon to Sheffield General Infirmary. J. T. Neech, L.R.C.P.Edin., D.S.Sc.Vict., appointed Physician to and Medical Superintendent of the Fever Hospital of the Leigh Joint Hospital Board. M. H. C. Palmer, L.R.C.P.Lond., L.R.C.S.Edin., appointed Medical Officer for the Second District of the Newbury Union. F. B. Reilly, L.R.C.P.Lond., M.R.C.S.Eng., appointed Medical Officer of Health to the Billinge Urban Sanitary District. J. J. Reynolds, L.R.C.P.Lond., M.R.C.S.Eng., appointed Medical Officer for the Cheriton Fitzpaine District of the Crediton Union. F. H. Roberts, L.R.C.P.Lond., M.R.C.S.Eng., appointed Medical Officer of Health for the Llandrindod Wells Urban Sanitary District. J. L. Russell, M.B., C.M.Edin., appointed Medical Officer for the Cornholme District of the Todmorden Union. F. O. Simpson, L.R.C.P., M.R.C.S., appointed Pathologist and Third Assistant Medical Officer to the West Riding Asylum, Wakefield. J. C. Smyth, M.R.C.S.Eng., L.R.C.P. Lond., appointed Medical Officer of Health to the Wells Rural District. J. A. E. Stuart, L.R.C.S., L.R.C.P.Edin., appointed Medical Officer of Health to the Batley Urban Sanitary District. A. E. Taylor, M.B., appointed Medical Officer for the Girlingham District of the Hartismere Union. J. W. Taylor, M.R.C.S., L.R.C.P.Lond., appointed Medical Officer of Health for the Methley Urban Sanitary District. F. Thomson, M.B.Aberd., D.P.H.Lond., appointed Temporary Medical Superintendent at the Gore Farm Hospital. A. F. Voelcker, M.R.C.S.Eng., L.S.A., appointed Assistant Physician to the Middlesex Hospital. Mr. E. Wakeham, appointed Medical Officer of Health for the Springhead Urban Sanitary District. F. H. Weekes, F.R.C.S.Eng., appointed Additional Honorary Medical Officer to the York Dispensary. E. Whichello, B.A., M.B., B.C.Cantab., appointed Assistant House Surgeon to the Huddersfield Infirmary. D. C. L. Williams, L.R.C.P., L.R.C.S.Edin., appointed Medical Officer for the Crowland District of the Peterborough Union. Dr. E. Williams, appointed Medical Officer of Health for the Penllyn Rural Sanitary District. Dr. Wilson, appointed Medical Officer of Health for the Standish Urban District.

### VACANCIES.

The following vacancies are announced this week, the amount of salary in each case being given after the name of the institution:—

### Resident Medical Officers.

Cumberland Infirmary, Carlisle.—House Surgeon. Salary £70 per annum, with board, lodging, and washing. Appointment for one year. Applications to the Secretary by August 24th.

Glamorgan County Asylum, near Bridgend.—Junior Assistant Medical Officer. Salary £130 per annum, rising £10 yearly to £150, with board (no beer or wine), lodging, and washing. Applications to the Medical Superintendent by August 22nd.

Jaffray Suburban Branch of the General Hospital, Gravelly Hill, Birmingham.—Resident Medical and Surgical Officer, doubly qualified. Salary £150 per annum, with board, residence, and washing. Applications to the House Governor, General Hospital, Birmingham, by August 24th.

Salisbury Infirmary.—House Surgeon, unmarried, doubly qualified. Salary £100 per annum, with board, lodging, and washing. Applications to the Secretary by August 23rd.

Teignmouth Infirmary, Dispensary, and Convalescent Home.—House Surgeon (to act also as Secretary), doubly qualified. Salary £60 per annum, with rooms, board, and washing. Applications to the Chairman of Committee by August 19th.

### Non-Resident Medical Officer.

Manchester Hospital for Consumption and Diseases of the Chest.—Honorary Assistant Physician. Must be on the *Medical Register*. Applications to Charles Behrens, Honorary Secretary, before August 20th.

Newcastle-on-Tyne Dispensary.—Visiting Medical Assistant, doubly qualified. Salary £120 for the first year, and £150 afterwards. Applications to R. W. Sisson, Honorary Secretary, by August 21st.

